# The sulfhydryl containing compounds WR-2721 and glutathione as radio- and chemoprotective agents. A review, indications for use and prospects

**DOI:** 10.1038/sj.bjc.6690404

**Published:** 1999-05

**Authors:** G A P Hospers, E A Eisenhauer, E G E de Vries

**Affiliations:** Division of Medical Oncology, Department of Internal Medicine, PO Box 30.001, Groningen, 9700 RB, The Netherlands; NCIC Clinical Trials Group, Queen's University, Kingston, Ontario, Canada

**Keywords:** WR-2721, glutathione, chemoprotection

## Abstract

Radio- and chemotherapy for the treatment of malignancies are often associated with significant toxicity. One approach to reduce the toxicity is the concomitant treatment with chemoprotective agents. This article reviews two sulfhydryl compounds, namely the agent WR-2721 (amifostine), a compound recently registered for use in human in many countries, and the natural occurring compound glutathione (GSH). GSH is not registered as a chemoprotective agent. WR-2721 is an aminothiol prodrug and has to be converted to the active compound WR-1065 by membrane-bound alkaline phosphatase. WR-1065 and GSH both act as naturally occurring thiols. No protective effect on the tumour has been found when these compounds are administered intravenously. There is even in vitro evidence for an increased anti-tumour effect with mafosfamide after pretreatment with WR-2721, and in vivo after treatment with carboplatin and paclitaxel. Randomized clinical studies have shown that WR-2721 and GSH decrease cisplatin-induced nephrotoxicity and that WR-2721 reduces radiation radiotherapy-induced toxicity. Side-effects associated with WR-2721 are nausea, vomiting and hypotension, GSH has no side-effects. An exact role of WR-2721 and GSH as chemoprotectors is not yet completely clear. Future studies should examine the protective effect of these drugs on mucositis, cardiac toxicity, neuro- and ototoxicity, the development of secondary neoplasms and their effect on quality of life. © 1999 Cancer Research Campaign


*British Journal of Cancer* (1999) **80**(5/6), 629–638
© 1999 Cancer Research Campaign
Article no. bjoc.1998.0404

### The sulfhydryl containing compounds WR-2721 and glutathione as radio- and chemoprotective agents. A review, indications for use and prospects

**GAP Hospers[1], EA Eisenhauer[2] and EGE de Vries[1]**

[1]Division of Medical Oncology, Department of Internal Medicine, University Hospital Groningen, PO Box 30.001, 9700 RB Groningen, The Netherlands; [2]NCIC Clinical Trials Group, Queen's University, Kingston, Ontario, Canada

**Summary** Radio- and chemotherapy for the treatment of malignancies are often associated with significant toxicity. One approach to reduce the toxicity is the concomitant treatment with chemoprotective agents. This article reviews two sulfhydryl compounds, namely the agent WR-2721 (amifostine), a compound recently registered for use in human in many countries, and the natural occurring compound glutathione (GSH). GSH is not registered as a chemoprotective agent. WR-2721 is an aminothiol prodrug and has to be converted to the active compound WR-1065 by membrane-bound alkaline phosphatase. WR-1065 and GSH both act as naturally occurring thiols. No protective effect on the tumour has been found when these compounds are administered intravenously. There is even in vitro evidence for an increased anti-tumour effect with mafosfamide after pretreatment with WR-2721, and in vivo after treatment with carboplatin and paclitaxel. Randomized clinical studies have shown that WR-2721 and GSH decrease cisplatin-induced nephrotoxicity and that WR-2721 reduces radiation radiotherapy-induced toxicity. Side-effects associated with WR-2721 are nausea, vomiting and hypotension, GSH has no side-effects. An exact role of WR-2721 and GSH as chemoprotectors is not yet completely clear. Future studies should examine the protective effect of these drugs on mucositis, cardiac toxicity, neuro- and ototoxicity, the development of secondary neoplasms and their effect on quality of life.

**Keywords:** WR-2721; glutathione; chemoprotection

In general, radio- or chemotherapy does not discriminate between normal and malignant cells and, for optimal anti-tumour effect, toxic effects on normal tissues often have to be accepted. For the majority of agents, myelosuppression is dose-limiting but all agents can produce significant non-haematologic effects which may limit individual doses (e.g. haemorrhagic cystitis for oxazophosphorine-based alkylating agents; neurotoxicity for taxoids; vomiting for cisplatin) and/or cumulative doses (e.g. neurotoxicity and nephrotoxicity for cisplatin; cardiac toxicity for anthracyclines). Fortunately, acute nausea and vomiting are better managed now than they were a decade ago, with the wide availability of 5-HT3 receptor antagonists, but other non-haematologic toxicities remain important problems. These toxicities are most pronounced when higher doses or longer term treatment is planned, but they can also occur when standard dose therapy is given, causing significant patient symptomatology and limiting the delivery of 'curative intent' treatment.

In general, several approaches are available which may prevent the development of serious toxicities, without necessarily sacrificing anti-tumour efficacy. These include: (1) changing the schedule of drug administration (e.g. infusion rather than bolus anthracyclines); (2) changing the route of drug administration (e.g. intraperitoneal cisplatin instead of intravenously (i.v.)); (3) biochemical modulation of the drug (e.g. administration of leucovorin 'rescue' with methotrexate); (4) addition of chemoprotectors (e.g. glutathione (GSH) or WR-2721 for cisplatin; dexrazoxane for anthracyclines; mesna for ifosfamide).

An ideal chemoprotective agent is an agent without side-effects, chemoprotective capacities and which does not reduce anti-tumour efficacy. Reducing the morbidity and mortality of our present antineoplastic regimens with the concomitant use of chemoprotective agents may make anti-tumour treatment more tolerable for patients and may permit for dose-escalation of radio- and chemotherapy, which could lead to improved survival.

WR-2721 (amifostine; Ethyol) and GSH are chemoprotective agents. WR-2721 is a synthetic aminothiophosphorate. GSH is an endogenous intracellular tripeptide thiol-containing compound. An important consideration in the study of chemoprotectors is the relative selectivity of the protective action, because these agents, theoretically, might also inhibit cytotoxic and anti-tumour activity. In vitro and in vivo experiments with WR-2721 did not show inhibition of anti-tumour activity (Yuhas, 1980; Calabro-Jones et al, 1988; Smoluk et al, 1988; Kemp et al, 1996). In fact, several in vitro and animal studies were able to demonstrate an increased cytotoxicity when tumours were exposed to chemotherapy combined with WR-2721 (Valeriote and Tolen, 1982; Millar et al, 1982; Treskes et al, 1994; Douay et al, 1995; Taylor et al, 1997). Intracellular GSH correlates in vitro and in vivo with an increase in resistance to chemotherapeutic drugs, but extracellular GSH (i.e. exogenous) does not reduce cytotoxic activity (Zunino et al, 1989; Leone et al, 1992; Hamers et al, 1993). In contrast to the locally active chemoprotective agent mesna (sodium 2-mercaptoethane sulphonate), which protects the bladder against cyclophosphamide-induced cystitis, GSH and WR-2721 are expected to protect more organs against chemo- and radiation

*Received 28 October 1998*
*Revised 9 November 1998*
*Accepted 10 November 1998*

*Correspondence to*: GAP Hospers therapy. Based on distribution studies of radiolabelled WR-2721, cytoprotection of kidney, heart, lung, liver, mucosa and bone marrow is predicted (Utley et al, 1976; Washburn et al, 1976). Based on the level of the enzyme, γ-glutamyl transpeptidase, necessary for the membrane transport of GSH and radiolabelled GSH, GSH is likely to have protective effects on kidney, liver and peripheral neurons (Romero et al, 1990; Hamers et al, 1993; Ercan et al, 1994).

In addition, the potential role of these agents in preventing mucosal, cardiac, oto- and neurotoxicity and in preventing the development of secondary malignancies in patients treated with radio- or chemotherapy will be discussed. Special attention will be paid to the characteristics, mechanisms of action, indication for use and the future prospects of these drugs.

### WR-2721 CHARACTERISTICS

WR-2721 (amifostine, (S-2-(3-aminopropylamino)-ethylphosphorothioic acid, Ethyol) was developed by the United States Army at the Walter Reed Army Institute of Research in the late 1950s, as part of the Anti-Radiation Drug Development Program to protect soldiers on the battlefield. This agent was selected from 4000 sulfhydryl compounds screened for further clinical evaluation because it was found to be one of the least toxic and most effective. Although it produced only slight effects in cultured cells as a single agent, the addition of alkaline phosphatase markedly increased the protective effects (Calabro-Jones et al, 1985; Mori et al, 1984). This was because WR-2721 is, in fact, a pro-drug which must be dephosphorylated to the active metabolite WR-1065 (Figure 1). The ability of WR-2721 to selectively protect normal tissues against both radiation and chemotherapeutic drugs is accounted for, in part, by higher uptake in normal as compared to tumour tissue (Calabro-Jones et al, 1985). This differential uptake is probably caused by differences in the microenvironment of alkaline phosphatase in normal and malignant tissue. High concentrations of this enzyme are found in capillaries and arterioles. In contrast, lower levels of alkaline phosphatase in capillaries of malignant tissue are common (Calabro-Jones et al, 1988). In addition, uptake of WR-1065 is highly dependent on pH. Because of their predominantly anaerobic metabolism, tumour tissues tend to have a lower pH than normal tissue. The decreased pH results in significantly reduced uptake of WR-1065 by tumour cells (Calabro-Jones et al, 1988). Also a difference in carrier-mediated

**Table 1** Comparative clinical studies with WR-2721

| Reference | Study design | Tumour type | No. of patients | Schedule of protectant | Antineoplastic regimen |
|---|---|---|---|---|---|
| Betticher et al (1995) | p,r | NSLC | W 11<br>C 10 | 910 or 683 mg $m^{-2}$ over 15 min prior and for 2 doses after CBDCA | CBDCA 600 mg $m^{-2}$ every 4 weeks for 4 cycles |
| Budd et al (1996) | p,r | NSCL | W 17<br>C 20 | 910 mg $m^{-2}$ over 15 min before and 2 hours after CBDCA | CBDCA 500 mg $m^{-2}$ every 4 weeks |
| Kemp et al (1996) | p,r | Ovar. | W 122<br>C 120 | 910 mg $m^{-2}$ over 15 min prior to CYC | CYC 1000 mg $m^{-2}$ then CDDP 100 mg $m^{-2}$ every 3 weeks for 6 cycles |
| Glick et al (1984) | co | Dif. | T 40 | 450–1100 mg $m^{-2}$ as a 15–20 mg $m^{-2}$ $min^{-1}$ infusion over 15 min prior to CYC | CYC 1200–1800 mg $m^{-2}$ |
| Glover et al (1986) | co | Ovar. | T 21 | 740 mg $m^{-2}$ over 15 min initiated 30 min prior to CYC | CYC 1500 mg $m^{-2}$ |
| Mollman et al (1988) | p | Dif. | W 28<br>C 41 | 740 mg $m^{-2}$ in those receiving CDDP<br>120 mg $m^{-2}$ monthly | CDDP 20–120 mg $m^{-2}$ and other various agents |
| Poplin et al (1994) | p,r | Rect. | W 48<br>C 49 | 910 mg $m^{-2}$ administered 15 min prior to M | M 20 mg $m^{-2}$ every 6 weeks |
| Wooley et al (1983) | co | Ovar. | T 10 | 250–1000 mg $m^{-2}$ 30 min before or divided dose 30 min before and 6 h after CYC | CYC 1000–1352 mg $m^{-2}$ |
| Planting et al (1996) | p,r | Head/Neck | W 36<br>C 38 | 740 mg $m^{-2}$ over 15 min prior to CDDP | CDDP 70 mg $m^{-2}$ 4 $week^{-1}$ for 6 weeks |
| Liu et al (1992) | p,r | Rect. | W 50<br>C 50 | 340 mg $m^{-2}$ over 15 min prior to every 2.25 Gy | Whole pelvis irrad. with 2.25 Gy 4 days/week for 5 weeks to a total of 45 Gy then 1 or 2 conedown of 7.20 Gy over 4 days |
| Brizel et al (1998) | p,r | Head/Neck | T 315 | 200 mg $m^{-2}$ 30 min before radiation | 1.8–2.0 Gy per day to total of 60–70 Gy |
| Buntzel et al (1998) | p,r | Head/Neck | W 25<br>C 14 | 500 mg prior to CBDCA | CBDCA 70 mg $m^{-2}$ day 1–5 and 21–25<br>Irrad. 2 Gy 5 days per week to a total of 60 Gy |

 *© Cancer Research Campaign 1999*

transport in tissues may play a role as seen for GSH (Hamers et al, 1993; Grdina et al, 1995). A difference not only in vascular supply but also in adhesion molecules and transport molecules in vessels might be an explanation for the preferential protection of tumour tissue (Nooijen et al, 1998).

In vitro and in vivo studies in animals have shown that WR-2721 provides selective protection of normal tissue against the toxicity of radiation and numerous chemotherapeutic agents, including alkylating agents, cisplatin, docetaxel and 5-fluorouracil (5-FU). In animal studies an increasing dose of WR-2721 resulted in better radioprotection. When given with cisplatin, the timing of WR-2721 administration might be important, because WR-2721 can form a complex with this drug very rapidly (Thompson et al, 1995). However, because 90% of the WR-2721 is cleared from the plasma within 6 min after administration of 740 mg $m^{-2}$ WR-2721 over 15 min, injection of cisplatin 15 min after WR-2721 administration can be carried out with no WR-2721 or metabolite detected. This permits both drugs to be given without complex formation (Shaw et al, 1986; Korst et al, 1997). When, however, 910 mg $m^{-2}$ WR-2721 is given there is evidence of saturable metabolism (Shaw et al, 1994). Fifteen minutes after the administration of WR-2721 there is still approximately 15% WR-2721 left in the plasma (range 1–30%, mean 20%). This remnant can form complexes with cisplatin. In carboplatin and paclitaxel in vitro and in vivo no adverse effect on tumour response is found when WR-2721 is given simultaneously (Taylor et al, 1997; Budd et al, 1996). Based on these results, WR-2721 drug complex formation might be not so important for these two drugs.

WR-2721 is unable to pass through the blood–brain barrier and cannot be used as a protective agent for the brain and spinal cord (Washburn et al, 1976; Millar et al, 1982). The drug-related toxicities in humans (Kemp et al, 1996) include hypotension (57%), flushing (39%), sneezing (25%), dizziness (11%) and chills (4%). Based on the side-effects, pharmacokinetic interactions and chemotherapeutic or radiotherapeutic schedule used, different concentrations and schedules of WR-2721 are applied. In some regimens, carboplatin is combined with three doses of amifostine because of the high concentration of the active carboplatin species during the first 4 h after administration. In general, for a short and frequent schedule (as radiotherapy), lower WR-2721 concentrations are used. The dose-limiting toxicity is hypotension. In the clinical setting, 200 mg $m^{-2}$ to 910 mg $m^{-2}$ WR-2721 administered in a 15-min infusion, is a tolerable dose when it is combined with anti-emetics and dexamethasone in the higher WR-2721

| Anti-tumour effect | Haematological protection (W vs C) | Non-haematological protection (W vs C) | Conclusion |
|---|---|---|---|
| Resp. 50% vs 22% (NS)<br>Med. Surv. 14 vs 9 month (NS) | NS | | No significant protection |
| Resp. NS | Incidence of granulocytopenia 12 vs 20%<br>Median nadir platelet count increased 72% | | Protection against haematological toxicity |
| Path. Resp. 43% vs 36%<br>Med Surv. 31 vs 31 month | Incidence of grade 4 neutropenia and fever: 10 vs 27% | Patients who discontinued therapy because of nephrotoxicity: 0 vs 8%<br>Neurotoxicity and ototoxicity not significantly different | Protection against haematological and non-haematological toxicity |
| | Granulocyte nodirs increased 67–88% | | Protection against the CYC induced haematological toxicity |
| | Mean nadir WBC count increased 42%<br>Mean nadir granulocyte count increased 130% | | Protection against CYC induced haematological toxicity |
| | | Incidence of neuropathy reduced: 25% vs 47–100% | Reduced CDDP induced neuropathy |
| Med Surv. 7.2 vs 6.3 months (NS) | WBC and platelet nadirs not significantly different | Data were not controlled | No significant protection |
| | WBC and platelet nadirs not significantly different between | treatments | No significant protection |
| Resp. 70% vs 57% | Platelet toxicity 3/4: 3% vs 17%<br>Treatment delay based on bone marrow toxicity 6% vs 21% | Ototoxicity 22% vs 34% | Protection against haematological toxicity |
| Resp. 16% vs 10% (NS) | | Significant difference in late severe toxicity | Protection against the late severe toxicity |
| Resp. 80% vs 78% (NS) | | Grade 2 xerostomia 50% vs 76%<br>onset grade 2 xerostomia 50 Gy vs 42 Gy | Protection against the acute severe toxicity |
| Resp. 79% vs 64% | Platelet toxicity 3/4: 0% vs 29% | Grade 3/4 mucositis 0% vs 86% grade 2 late xerostomia 17% vs 55% | Protection against the acute and late toxicity |

*© Cancer Research Campaign 1999*

**Table 2** Comparative clinical studies with GSH

| Reference | Study design | Tumour type | No. of patients | Schedule of protectant | Antineoplastic regimen | Anti-tumour effect | Haematological protection (G vs C) | Non -haematological protection (P vs C) | Conclusion |
|---|---|---|---|---|---|---|---|---|---|
| Parnis et al (1995) | p,r | Ovar. | G 6<br>C 6 | 1500 mg $m^{-2}$ over 15 min prior to CDDP | CDDP 40 mg $m^{-2}$ administered over 2 h for respectively 2, 3 or 4 successive days, in each group two patients | | No significant difference | No significant difference | No significant protection |
| Smyth et al (1997) | p,r | Ovar. | G 74<br>C 77 | 3000 mg $m^{-2}$ over 15 min prior to CDDP | CDDP 100 mg $m^{-2}$ | Resp. 73% vs 62% | | Significant difference in nephrotoxicity | Protection against renal toxicity |
| Bogliun et al (1996) | p,r | Ovar. | G 27<br>C 27 | 2500 mg $m^{-2}$ over 15 min prior to CDDP | CDDP 50 mg $m^{-2}$ in 26 patients (G 12, C 14)<br>CDDP 75 mg $m^{-2}$ in 28 patients (G 15, C 13) | Resp. 72% vs 52% | No significant difference | No significant difference | |

p = parallel study, r = randomized study, co = cross-over study, T = total, W = WR-2721, C = control, CBDCA = = carboplatin, CDDP = cisplatin, CYC = cyclophosphamide, M = melphalan, WBC = white blood cells, NS = not significant, NSCLC = non-small-cell lung cancer, Ovar. = ovarian, Dif. = different tumours, Rect. = Rectal, Path. Resp. = Pathological Response, Resp. = Response Rate, Med Surv. = Median Survival., * = caused by different amount of drugs.

$$NH_2(CH_2)_3\text{-}NH(CH_2)_2\text{-}S\text{-}PO_3H_2 \xrightarrow{} NH_2(CH_2)_3\text{-}NH(CH_2)_2\text{-}SH$$

WR-2721 AF WR-1065

**Figure 1** WR-2721 is converted to the active dephosphorylated metabolite (WR-1065) by the enzyme alkaline phosphatase (AF)

concentrations. Premedication with 20 mg dexamethasone intravenously and a serotonin receptor antagonist modifies these effects so that only 1% of infusions are associated with WHO grade 3/4 vomiting and less than 1% of patients require infusion interruption for hypotension (Planting et al, 1996). No dexamethasone premedication is necessary at a dose of 200 mg $m^{-2}$ amifostine, as used in radiotherapy applications. This drug is registered in many countries for the prevention of cisplatin-induced nephrotoxicity. Beside the indications as a chemoprotective agent, WR-2721 is investigated as a bone marrow stimulant in patients with myelodysplastic syndrome. At a dose of 200 mg $m^{-2}$ three times a week, haematological activity is shown (List et al, 1997). The US Federal Drug Agency (FDA) recommended dose for chemoprotection in adults is 910 mg $m^{-2}$ administered as a 15-min infusion 30 min before the start of the chemotherapy. The price of 500 mg WR-2721 in the Netherlands is approximately US $250. Per chemotherapy course this would mean an additional expense of approximately US $650.

### GSH CHARACTERISTICS

GSH is a naturally occurring tripeptide (γ-glutamine–cysteine–glycine) and the most abundant intracellular thiol (Figure 2). Although all tissues produce GSH, some synthesize more than others. The liver, for instance, is a net exporter of this peptide. Exogenously administered GSH is reported to increase intracellular GSH levels, but this increase is not caused by the uptake of exogenously administered GSH (Meister, 1983). This intracellular uptake of GSH from plasma almost always involves its degradation by membrane-bound γ-glutamyl transpeptidase, followed by transport into the cell of the individual amino acids and resynthesis of GSH by γ-glutamylcysteine synthetase and glutathione synthetase. Glutamate is coupled by γ-glutamyl transferase to another amino acid, and this dipeptide is transported across the cell membrane (Figure 3). Glutathione synthetase is not inhibited by glutathione, therefore higher intracellular GSH concentrations can be reached (Meister et al, 1983). Organs such as kidney, liver and peripheral nerves containing high levels of transpeptidase activity are important scavengers of plasma GSH. The kidney is the most important scavenger, but peripheral nerves also contain quite high levels of γ-glutamyl transpeptidase, indicating they are also capable of importing exogenous GSH. The selective protection of GSH for normal compared to tumour cells is likely related to low membrane γ-glutamyl transpeptidase activity in tumour cells (Hamers et al, 1993). The organs listed containing a high level of γ-glutamyl transpeptidase activity are those which theoretically could be protected by exogenous GSH administration following radiation or chemotherapy. The half-life of GSH is approximately 15 min after 2000 mg $m^{-2}$ GSH. In 90 min following infusion, the urinary excretion of GSH and cysteine was increased 300-fold and tenfold respectively (Aebi et al, 1991). Compared with a lower dose, the disposition of GSH appears to be dose-dependent and subject to saturation kinetics. Theoretically, the timing of the start

*© Cancer Research Campaign 1999*

**Table 3** Ongoing clinical trials using WR-2721 (PDQ clinical trial search)

| Study design | Tumour type | Antineoplastic regimen | No. of studies |
|---|---|---|---|
| Phase I/II | Myelodysplastic syndrome | WR-2721 | 6 |
| Phase II | Breast, NSCLC | Paclitaxel | 3 |
| Phase I/II | NSCLC, ovar., endometria adenocarcinomal | Paclitaxel and CDDP or CBDCA | 7 |
| Phase I/II | Colorect. | Irinotecan | 1 |
| Phase III | Dif. | CDDP | 1 |
| Phase I/II | Dif. | CDDP and Gemcitabine | 2 |
| Phase I | Acute myeloid leukaemia | Cytarabine and Mitoxantrone | 1 |
| Phase I | Dif | Ifosfamide and CBDCA and Etoposide | 1 |
| Phase II | Breast | Mitoxantrone and Thiotepa and CYC | 1 |
| | Head and Neck | 5-FU and CDDP | 1 |
| Phase I/II | | Fludarabine | 1 |

CBDCA = carboplatin, CDDP = cisplatin, CYC = cyclophosphamide NSCLC = non-small-cell lung cancer, Ovar. = ovarian, Dif. = different tumours, Rect. = Rectal.

of the GSH infusion before the cisplatin might be critical because, like WR-2721, GSH can form a complex with cisplatin. When cisplatin is given 30–60 min after the GSH infusion in a ratio of GSH:CDDP of 30:1 there is protection without interfering with therapeutic activity (Zunino et al, 1989). In order to maximize the cytoprotective effect of GSH, short cisplatin infusions should be given. In the clinical setting 1500–3000 mg $m^{-2}$ GSH is administered i.v. approximately 15 min before chemotherapy (Parnis et al, 1995; Smyth et al, 1997; Bogliun et al, 1996). In contrast with WR-2721, GSH itself produces no toxicity. This drug is not registered as a chemoprotective agent. In the USA, GSH is also being studied in patients with HIV infection to assess the potential benefit of increasing the abnormally low blood GSH levels found in this setting. In addition, the cytoprotective potential of this drug is being studied with both chemotherapy and radiotherapy in several clinical studies (Di Re et al, 1990, 1993; Hamers et al, 1993; Locatelli et al, 1993).

### MECHANISMS OF PROTECTIVE ACTION OF WR-1065 AND GSH

WR-1065 and GSH act as naturally occurring thiols. They prevent cell damage by scavenging of hydroxyl radicals, the chemical repair of DNA radicals by hydrogen atom transfer, the depletion of oxygen as a consequence of thiol oxidation, the protection of key sulfhydryl enzymes through formation of protein–aminothiol mixed disulphides, and the facilitation of DNA repair through binding of the disulphide to DNA resulting in stabilization of DNA and inhibition of replication. WR-2721 has a similarity in structure to polyamines and, also like polyamines, has a high affinity for DNA and polyamine effects on processes related to DNA structure and synthesis (Murley and Grdina, 1995; Murley et al, 1997; Weiss, 1997). WR-1065 can give, in concentrations from 4 μM in Chinese hamster ovary cells, a delay in the cell cycle and an inhibition of topoisomerase IIα activity (Murley et al, 1997). Based on the above described mechanisms, WR-1065 and GSH act as cytoprotective agents following radiation and with chemotherapeutic drugs such as alkylating agents, cisplatin and doxorubicin. The mechanisms for protection of radiation effects by these drugs occur probably by scavenging free radicals, reaction with alkyl groups, prevention of platinum-adduct formation and a faster DNA repair (Treskes et al, 1991). Preclinical and clinical studies have shown that WR-2721 also provides selective protection of normal tissue against the toxicity of paclitaxel, docetaxel and 5-FU (Wasserman et al, 1981). In a non-randomized trial, it appeared that amifostine pretreatment has the tendency to reduce the paclitaxel-induced arthralgia, myalgia, myelosuppression and neuropathy (DiPaola et al, 1998). The mechanism(s) by which it (they) protects against paclitaxel, docetaxel and 5-FU toxicity is not known, or even if they protect in the clinic. In addition, confirmatory randomized data are required before it can be stated that amifostine provides protection for the effects of these agents in the clinic. It may be that a faster DNA repair is responsible for the protective activity of 5-FU.

### CLINICAL RESULTS OF RADIO- AND CHEMOTHERAPY PROTECTION BY WR-2721

In Table 1, different comparative clinical trials using WR-2721 as radio- and chemoprotective agent are presented; unfortunately, most studies are small. Most comparative trials showed WR-2721 to reduce the severity and/or incidence of granulocytopenia and thrombocytopenia observed after cisplatin (CDDP) plus cyclophosphamide or cyclophosphamide alone compared with a control group or in patients who crossed to chemotherapy alone. The largest randomized trial is that of Kemp et al (1996). In total, 242 patients with advanced epithelial ovarian cancer were randomized to treatment with cyclophosphamide (1000 mg $m^{-2}$) plus CDDP (100 mg $m^{-2}$) with or without amifostine (910 mg $m^{-2}$), every 3 weeks for 6 cycles. WR-2721 was shown to reduce the renal toxicity from cisplatin given in a dose of 100 mg $m^{-2}$ CDDP. The survival in the two groups was similar, providing reassurance that WR-2721 did not cause tumour protection. As a result of the reduction in renal, haematologic and neurotoxicity only 9% of patients in the WR-2721 arm discontinued chemotherapy, compared with 24% in the control arm.

Liu et al (1992) published a randomized study in 100 patients with radiotherapy (entire pelvis: 4500 cGy, conedown: 720 cGy) with or without WR-2721 (340 mg $m^{-2}$) in patients with advanced rectal cancer. The radiation toxicity was scored on the Radiation Therapy Oncology Group (RTOG) scale. A reaction was considered acute if it occurred within 105 days after the first radiotherapy day. No moderate or severe late toxicity from radiation was seen in the normal tissues in patients who received WR-2721. Five patients

© Cancer Research Campaign 1999 

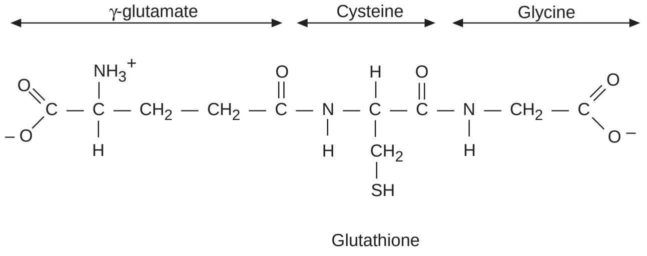

**Figure 2** Structure of GSH

treated with radiotherapy alone had a moderate or severe toxicity to one or more of the following organs: skin, genitourinary or lower gastrointestinal tract. The WR-2721 was administered i.v. before each radiotherapy session. Although there was a significant reduction in late radiation-induced toxicity, there was no effect on acute toxicity in this group of rectal cancer patients. There was no evidence of tumour protection by WR-2721: 16% of the patients randomized to the WR-2721 group had a complete response compared with 10% in the group with radiotherapy only. This difference is, however, not significant because of the small numbers in each group; this is due to the fact that only 60% of the patients were evaluable. In a phase II study (Buntzel et al, 1998) in patients with head and neck cancer treated with radiotherapy and carboplatin, WR-2721 was administered i.v. in 25 patients compared with 14 patients in the control group. A reduced platelet toxicity and mucositis was found in the WR-2721-treated group. Brizel et al (1998) showed a reduction in the acute radiation-induced toxicity (xerostomia) in patients with head and neck cancer in the WR-2721-treated group compared with the control group.

To balance the overall effect of WR-2721, one has to take in toaccount, on one hand, the reduction in radiotherapy-induced toxicity and a small reduction of haematological and renal toxicity, and on the other hand, the side-effects of the WR-2721 infusion. However, with an improved premedication regimen, WR-2721 is tolerable.

### CLINICAL RESULTS OF CHEMOTHERAPY PROTECTION BY GSH

In Table 2 different comparative clinical trials using GSH as a chemoprotective agent are presented. For GSH, a protection of cisplatin-induced toxicity is noted in ovarian cancer comparable with that produced by WR-2721 (Smyth et al, 1997). In this randomized, double-blind study, 151 patients with ovarian cancer were treated with 100 mg $m^{-2}$ with or without GSH (3 g $m^{-2}$), every 3 weeks for 6 cycles. Nephrotoxicity occurred in 39% who received GSH versus 49% controls, and caused failure to complete protocol in 11/74 versus 26/77. In addition, quality of life was assessed, which showed a significant reduction in depression in the GSH-treated group. No tumour protection was found.

A small, randomized study with cisplatin with and without GSH has been published by Boglium et al (1996). In this study, 54 patients with ovarian cancer were treated with 50 or 75 mg $m^{-2}$ CDDP every 3 weeks for 9 or 6 courses respectively, with or without 2.5 g $m^{-2}$ GSH. In a small group with a cumulative dose of 500–675 mg $m^{-2}$ (ten patients without GSH and 13 patients with GSH), there was a trend toward less severe neurotoxicity. No difference in non-neurological side-effects were found. It can be concluded that, taken in account with the results of the phase II studies, GSH shows protective activity against cisplatin-induced renal toxicity.

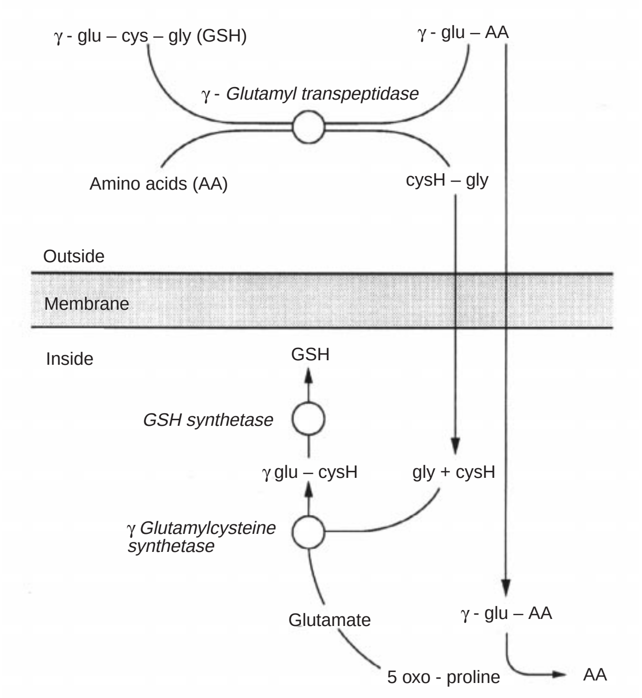

**Figure 3** The intracellular 'uptake' of GSH

In Table 3, ongoing clinical trials with WR-2721 are listed (PDQ clinical trial search), no trials are found with GSH in the PDQ clinical trial search. For the future it would be interesting to study the effect on the quality of life of both drugs (WR-2721 and GSH) after chemotherapeutic and radiation regimens. An increase in quality of life is expected when it is possible to prevent mucositis, neurotoxicity, ototoxicity, cardiac failure and the development of secondary tumours. For evaluation of protection, objective tests are necessary.

### DIFFERENCES BETWEEN THE CHEMOPROTECTIVE EFFECTS OF WR-2721 AND GSH

The basic structure of both sulfhydryl-containing drugs is completely different. The polyamine-like structure of WR-2721 suggests it may have a greater protective effect on DNA. Different organs are protected by WR-2721 and GSH. Based on the distribution studies of radioactive WR-2721 and GSH (Washburn et al, 1976; Ercan et al, 1994), radio- and chemotherapy protection for kidney, heart, lung, mucosa, liver and bone marrow is expected for WR-2721, and for GSH, protection is expected for kidney, liver and urinary bladder. Based on the level of the enzyme, γ-glutamyl transpeptidase necessary for the membrane transport of GSH, and according to animal data, GSH is likely to have a protective effect on peripheral neurons (Romero et al, 1990; Hamers et al, 1993).

Based on the available laboratory data, WR-2721 shows protection against radiotherapy, cisplatin, alkylating agents, 5-FU and taxanes, and GSH shows protection against alkylating agents and cisplatin.

 © Cancer Research Campaign 1999

Clinical data on all except radiation, alkylation and cisplatin/ CBDCA are weak. Clinical data from WR-2721 and GSH show protection against renal and bone marrow toxicity. Importantly, GSH can supply this protection without major side-effects. However, on the above mentioned preclinical data, WR-2721 has, theoretically, an extended clinical application.

### FUTURE PROSPECTS

### Local effects on mucous- and visceral membranes

Oral mucositis and rectal wall injury are important chemo- and radiation treatment-related toxicities. Local application of WR-2721 to the small bowel mucosa of rats before radiation provides protection and this benefit is amplified when WR-2721 is in an alkaline medium (Delaney et al, 1994). Local application of WR-2721 rectally in 21 patients prior to each fraction of pelvic radiation did not show a protective effect when compared to historical controls treated without WR-2721. This was in spite of a high concentration of alkaline phosphatase in the rectal wall (Ben-Josef et al, 1995). The reasons for failure of this drug to protect the rectosigmoid mucosa may be related to the method of administration: the rectum was not empty, only 30 ml were given and radiation was given until 45 min later (Montana et al, 1992). In a phase II study in patients with head and neck cancer having local radiotherapy, less mucositis was found in the group that received WR-2721 i.v. (Buntzel et al, 1998). Prevention of oral mucositis with local application of WR-2721 has not been investigated to date. The taste of WR-2721 (20 mg $ml^{-1}$) is acceptable (personal observation), but it is uncertain if the activity of alkaline phosphatase in the mouth or saliva is sufficient to produce WR-2721 to the active WR-1065 component. Alkaline phosphatase is present in the oral mucosa, but the concentration is ten times lower than that of the rectal mucosa. However, the concentration of alkaline phosphatase is probably not the only factor necessary to convert WR-2721 because, based on in vitro experiments, myocytes have a low degree of alkaline phosphatase but can convert WR-2127 to the active compound (Dorr, 1996). Local application of GSH is also interesting to investigate, although the effect is questionable because the γ-glutamyl-transferase concentration in the oral mucosa and saliva is low (Sajjan et al, 1991; Schwint et al, 1992).

Recently, Alberts et al (1996) showed, in ovarian cancer, that intraperitoneal (i.p.) cisplatin significantly improves survival and has overall a significantly lower toxicity compared to i.v. cisplatin, but in the i.p. group an increase of abdominal pain and dyspnoea was found. Given WR-2721 i.v., only 1% of the dose appears in the ascites (Van der Vijgh and Kerst, 1996), therefore WR-2721 might be considered for i.p. administration to circumvent intra-abdominal distress. Based on monkey experiments of i.p. WR-2721, where 100% of the radioactive WR-2721 was found systemically with only a delay of approximately 10 min compared with i.v. administration (Mangold et al, 1989), it is likely that side-effects of i.p. WR-2721 would be similar to i.v. administration.

### Neurotoxicity and ototoxicity

Neurotoxicity and ototoxicity are important irreversible problems in some curatively treated patients, notably those with germ cell tumours. Neuropathy has been reported to occur in 30–100% of patients with germ cell tumours treated with cisplatin and is irreversible in 30–50% of the patients (Alberts and Noel, 1995; De Wit, 1995). A trial of WR-2721 in this group of patients has not been undertaken, perhaps because of concern over tumour protection in this curable disease and the knowledge that testicular germ cell tumours can express alkaline phosphatases. However, in an animal model with an alkaline phosphatase-positive tumour, no evidence is found for tumour protection by WR-2721 (Dunn et al, 1996). In Wistar rats, cisplatin-induced neuropathy was reduced after a dose of 200 mg GSH per kg body weight (Hamers et al, 1993). In hamsters, WR-2721 did not show any chemoprotection in combination with cisplatin on the cochlea (Kaltenbach et al, 1997). However, in a phase I study with high-dose cisplatin, radiotherapy and WR-2721 compared with a control group, there is an indication of a decrease in ototoxicity (Rubin et al, 1995). Recently (McGuire et al, 1996), the combination of cisplatin and paclitaxel has been shown to be very active in ovarian cancer, with an increase in median survival of 1.5 years, compared to the standard regimen of cyclophosphamide and cisplatin. Since both cisplatin and paclitaxel are known to be neurotoxic, the use of effective chemoprotective agents may increase the quality of life in patients treated with this regimen. Investigation of thiol compounds with this combination would therefore be of interest. Sensitive objective tests of neurologic function will be important to accurately document the impact of cytoprotective agents in this setting.

### Cardiac failure

Cardiac failure after chemo- and radiotherapy may arise as a late complication 5–10 years after treatment, often occurring in the subset of relatively young patients successfully ‘cured of the cancer’ (Lipschulz et al, 1991; Steinherz and Yahalon, 1993; Gyenes et al, 1996; Shapiro and Henderson, 1994). Dexrazoxane (ICRF-187, Zinecard, ADR-529), another chemoprotective agent, has been shown to significantly decrease anthracycline-related cardiotoxicity (Swain et al, 1997), without a decrease in survival. However, the patients treated had metastatic breast cancer with a median survival of only 5 months. There are only a few publications of the effect of thiols in this setting (Dorr, 1996). In vitro experiments with cardiac myocytes showed a decrease in the doxorubicin-related toxicity after incubation with WR-2721. An interesting observation in the WR-2721-treated cells was the detection of an increase in the GSH content (Dorr, 1996). No clinical studies with WR-2721 and GSH as protectors of radio- and chemotherapy-induced cardiac toxicity have been carried out. Cardiac failure after chemotherapy is often a late symptom. Therefore, objective early tests to demonstrate cardiac dysfunction have to be developed to evaluate the effect of protective agents at an early stage. In order to develop objective tests to evaluate cardiotoxicity, an awareness of different types of cardiotoxicity caused by chemo- and radiotherapy is required. Cardiotoxicity can be divided into three types of toxicity: (1) due to myocardial damage, (2) due to vascular damage, (3) due to activation- and conduction system damage (neurotoxicity). For the myocardial damage, a MUGA scan is a reasonable objective test. For the other two types of toxicities, objective early tests have to be developed.

### Therapy-induced secondary cancer

The use of radio- and chemotherapy has placed a large number of patients at potential risk for developing a treatment-related malignancy. On the other hand, it is becoming more apparent that host factors, for example a germline mutation in p53, can also increase the risk of developing a second tumour.

© Cancer Research Campaign 1999

In vitro WR-1065 showed a reduction in radio- and chemotherapy-induced mutation in hamster cells (Hill et al, 1986; Nagy and Grdina, 1986; Nagy et al, 1986), and reduced the proto-oncogene c-*myc* expression (Liu et al, 1997) and delayed a cell cycle progression and build-up cells in the G2 phase of the cell cycle (Murley et al, 1997). Pretreatment with amifostine reduced mutation frequency in cells from mice given radiation, cisplatin or cyclophosphamide (Kataoka et al, 1992; Grdina et al, 1992). WR-2721 has the ability to protect against radiation carcinogenesis. In mice treated with WR-2721, 26% of radiation-induced sarcomas developed compared with 87% in the mice exposed to radiation only (Milas et al, 1984). In the clinical setting, an advantage of WR-2721 and GSH may therefore be their potential to reduce the development of radio- and chemotherapy-induced secondary tumours.

### CONCLUSION

WR-2721 can provide selective protection against platinum and alkylating agents related to renal- and bone marrow toxicity and radiation-induced toxicity. Based on the randomized study of Smyth et al (1997) and phase II studies, GSH has the same protective profile as WR-2721 when given prior to cisplatin. GSH has no significant side-effects and is therefore a potential alternative for WR-2721. An important remark concerning these radio- and chemotherapy protective agents is that, when given extracellularly, no tumour resistance to radio- and chemotherapy has been noticed. Although these drugs show protection against chemotherapy-related renal- and bone marrow toxicity, and WR-2721 shows a protection against the toxicity of radiotherapy, the magnitude of this protection has no impact on the standard chemotherapeutic regimens. New studies should focus on the magnitude of clinical cytoprotection and the real clinical benefit. These agents may be useful in increasing the quality of life by decreasing mucositis, neurotoxicity, ototoxicity and cardiac toxicity, but this is speculative and studies to confirm this are needed. For preventing mucositis without the side-effect of systemically administered WR-2721, local application would be interesting to study.

### REFERENCES

Aebi S, Assereto R and Lauterburg BH (1991) High-dose intravenous glutathione in man. Pharmacokinetics and effects on cyst(e)ine in plasma and urine. *Eur J Clin Invest* **21**: 103–110

Alberts DS and Noel JK (1995) Cisplatin associated neurotoxicity, can it be prevented? *Anticancer Drugs* **6**: 369–383

Alberts DS, Liu PY, Hanigan EV, O'Toole R, Williams SD, Young JA, Franklin EW, Clarke-Pearson DL, Malviya VK, Dubeshter B, Adelson MD and Hoskins WJ (1996) Intraperitoneal cisplatin plus intravenous cyclophosphamide versus intravenous cisplatin plus intravenous cyclophosphamide for stage III ovarian cancer. *N Engl J Med* **335**: 1950–1956

Ben-Josef E, Mesina J, Shaw LM, Bonner HS, Shamsa F and Porter AT (1995) Topical application of WR-2721 achieves high concentrations in the rectal wall. *Radiation Res* **143**: 107–110

Betticher DC, Anderson H, Ranson M, Meeley K, Oster W and Thatcher N (1995) Carboplatin combined with amifostine, a bone marrow protectant, in the treatment of non-small cell lung cancer. A randomized phase II study. *Br J Cancer* **72**: 1551–1555

Bogliun G, Marzorati L, Marzola M, Miceli MD, Cantu MG and Cavaletti G (1996) Neurotoxicity of cisplatin +/− reduced glutathione in the first-line treatment of advanced ovarian cancer. *Int J Gynaecol Cancer* **6**: 415–419

Brizel D, Sauer M, Wannenmacher M, Henke M, Eschwege F and Wasserman T (1998) Randomized phase III trial of radiation +/− amifostine in patients with head and neck cancer. *Proc Am Soc Clin Oncol* **17**: Abstract # 1487.

Budd GT (1996) Amifostine and chemotherapy-related thrombocytopenia. *Semin Oncol* **23**: 49–52

Budd GT, Bukowski RM, Adelstein D, Pelley R, Olencki T, Petrus JV, McLain D, Conlon J, Kurman M, Capizzi RL and Ganapathi R (1996) Mature results of a randomized trial of carboplatin and amifostine vs carboplatin alone in patients with advanced malignancies. *Proc Am Soc Clin Oncol* **15**: (abstract) 1720

Buntzel J, Kuttner K, Frohlich D and Glatzel M (1998) Selective cytoprotection with amifostine in concurrent radiochemotherapy for head and neck cancer. *Ann Oncol* **9**: 505–509

Calabro-Jones PM, Fahey RC, Smoluk GD and Ward JF (1985) Alkaline phosphatase promotes radioprotection and accumulation of WR-1065 in V79–171 cells incubated in medium containing WR-2721. *Int J Radiat Biol* **47**: 23–27

Calabro-Jones PM, Aguilera JA, Ward JF, Smoluk GD and Fahey RC (1988) Uptake of WR-2721 derivatives by cells in culture: identification of the transported form of the drug. *Cancer Res* **48**: 3634–3640

Capizzi RL and Oster W (1995) Protection of normal tissues from the cytotoxic effects of chemotherapy and radiation by amifostine: clinical experience. *Eur J Cancer* **31A**: 8–13

Delaney JP, Bonsack ME and Felemovicius I (1994) Radioprotection of the rat small intestine with topical WR-2721. *Cancer* **74**: 2379–2384

De Wit R (1995) Four cycles of BEP versus an altering regime of PVB and BEP in patients with poor prognosis metastatic testicular non-seminoma. A randomised study of the EORTC Genitourinary Tract Cancer Cooperative Group. *Br J Cancer* **71**: 1311–1314

DiPaola RS, Rodriguez R, Goodin S, Recio A, Orlick M, Mollman J, Bird S, Belsh JM, Schein PS, Aisner J, Schuchter LM (1998) Amifostine and dose-intense paclitaxel in patients with advanced malignancies. *Cancer Therapeutics* **1**: 11–17

Di Re F, Bohm S, Oriana S, Spatti GB and Zunino F (1990) Efficacy and safety of high-dose cisplatin and cyclophosphamide with glutathione protection in the treatment of bulky advanced epithelial ovarian cancer. *Cancer Chemother Pharmacol* **25**: 355–360

Di Re F, Bohm S, Oriana S, Spatti GB, Pirovano C, Tedeschi M and Zunino F (1993) High-dose cisplatin and cyclophosphamide with glutathione in the treatment of advanced ovarian cancer. *Ann Oncol* **4**: 55–61

Dorr RT (1996) Cytoprotective agents for anthracyclines. *Semin Oncol* **23**: 23–24

Douay L, Hu C, Giarratana M-C, Bouchet S, Conlon J, Capizzi RL and Gorin N-C (1995) Amifostine improves the antileukemic therapeutic index of mafosfamide: implications for bone marrow purging. *Blood* **86**: 2849–2855

Dunn TA, Schmoll HJ, Grunwald V, Bokemeyer C and Casper J (1996) Amifostine does not alter the antitumor activity of cisplatin in a pre-clinical model of testicular cancer. *Anticancer Drugs* **7**: 795–799

Ercan MT, Aras T, Aktas A, Kaya S and Bekdik CF (1994) Accumulation of 99mTc-glutathione in head and neck tumors. *Nuklearmedizin* **33**: 224–228

Glick JH, Glover DJ and Weiler C (1984) Phase I controlled trials of WR-2721 and cyclophosphamide. *Int J Oncol Biol Phys* **10**: 1777–1780

Glover D, Glick JH, Weiler C, Fox K, Turrisi A and Klingerman MM (1986) Phase I/II trials of WR-2721 and cis-platinum. *Int J Radiat Oncol Biol Phys* **12**: 1509–1512

Grdina DJ, Kataoka Y and Basic I (1992) The radioprotector WR-2721 reduces neutron-induced mutations at the hypoxanthine-guanine phosphoribosyl transferase locus in mouse splenocytes when administered prior to or following irradiation. *Carcinogenesis* **13**: 811–813

Grdina DJ, Shigematsu N, Dale P, Nemton GL, Aguilera JA and Fahey RC (1995) Thiol and disulfide metabolites of the radiation protector and potential chemopreventive agent WR-2721 are linked to both its anti-cytotoxic and anti-mutagenic mechanisms of action. *Carcinogenesis* **16**: 767–774

Gyenes G, Fornander T, Carlens P and Skovsgaard T (1996) Morbidity of ischemic heart disease in early breast cancer 15–20 years after adjuvant radiotherapy. *Int J Rad Oncol Biol Phys* **28**: 1235–1241

Hamers FPT, Brakkee JH, Cavalletti E, Tedeschi M, Marmonti L, Pezzoni G, Neijt JP and Gispen WH (1993) Reduced glutathione protects against cisplatin-induced neurotoxicity in rats. *Cancer Res* **53**: 544–549

Hill CK, Nagy B and Persaino C (1986) WR-2721 is anti-neoplastic and anti-mutagenic when given during $^{60}$Co γ-ray irradiation. *Carcinogenesis* **7**: 665–668

Kaltenbach JA, Church MW, Blakley BW, McCaslin DL and Burgio DL (1997) Comparison of five agents in protecting the cochlea against the ototoxic effects of cisplatin in the hamster. *Otolaryngol Head Neck Surg* **117**: 493–500

Kataoka Y, Basic I and Perrin J (1992) Antimutagenic effects of radioprotector WR-2721 against fission spectrum neutrons and $^{60}$Co γ-rays in mice. *Int J Radiat Biol* **61**: 387–392

Kemp G, Rose P, Lurain J, Berman M, Manetta A, Roullet B, Homesley H, Belpomme D and Glick J (1996) Amifostine pretreatment for protection against cyclophosphamide-induced and cisplatin-induced toxicities: results of a randomized controlled trial in patients with advanced ovarian cancer. *J Clin Oncol* **14**: 2101–2112

© Cancer Research Campaign 1999

Korst AEC, Eeltink CM, Vermorken JB and Van de Vijgh WJF (1997) Pharmacokinetics of amifostine and its metabolites in patients. *Eur J Cancer* **33**: 1425–1429

Leone R, Fracasso ME, Soresi E, Cimino G, Tedeschi M, Castoldi D, Monzani V, Colombi L, Usari T and Bernareggi A (1992) Influence of glutathione administration on the disposition of free and total platinum in patients after administration of cisplatin. *Cancer Chemother Pharmacol* **29**: 385–390

Lipschulz SE, Colan SD, Gelber RD, Peres-Atayde AR, Sallan and Sanders SP (1991) Late cardiac effects of doxorubicin therapy for acute lymphoblastic leukaemia in childhood. *N Engl J Med* **324**: 808–815

List AF, Brasfield F, Heaton R, Glinsmann-Gibson B, Crook L, Taetle R and Capizzi R (1997) Stimulation of hematopoiesis by amifostine in patients with myelodysplastic syndrome. *Blood* **90**: 3364–3369

Liu TF, Liu Y, He S, Zhang Z and Kligerman MM (1992) The use of radiation with or without WR-2721 in advanced rectal cancer. *Cancer* **69**: 2820–2825

Liu SC, Murley JS, Woloschak G and Grdina DJ (1997) Repression of c-myc gene expression by the thiol and disulfide forms of the cytoprotector amifostine. *Carcinogenesis* **18**: 2457–2459

Locatelli MC, D'Antona A, Labianca R, Vinci M, Tedeschi M, Carcione R, Corbo A, Venturino P and Luporini G (1993) A phase II study of combination chemotherapy in advanced ovarian carcinoma with cisplatin and cyclophosphamide plus reduced glutathione as potential protective agent against cisplatin toxicity. *Tumori* **79**: 37–39

McGuire WP, Hoskins WJ, Brady MF, Kucera PR, Partridge EE, Look KY, Clarke-Pearson DL and Davidson M (1996) Cyclophosphamide and cisplatin compared with taxol and cisplatin in patients with stage III and stage IV ovarian cancer. *N Engl J Med* **33**: 1–6

Mangold DJ, Miller MA, Huelle BK, Sanchez-Barona DO, Swynnerton NF, Flechenstein L and Ludden TM (1989) Disposition of the radioprotector ethiofos in the rhesus monkey. Influence of route of administration. *Drug Metab Dispos* **17**: 304–310

Meister A (1983) Selective modification of glutathione metabolism. *Science* **220**: 472–477

Milas L, Hunter N, Stephens LC and Peters LJ (1984) Inhibition of radiation carcinogenesis in mice by S-2-(3-aminopropylamino)-ethylphosphorothioic acid). *Cancer Res* **44**: 5567–5569

Millar JL, McElwain TJ, Clutterbuck RD and Wist EA (1982) The modification of melphalan toxicity in tumour bearing mice by WR-2721. *Am J Clin Oncol* **5**: 321–328

Mollman JE, Glover DJ, Hogan WM and Furman RE (1988) Cisplatin neuropathy. Risk factors, prognosis, and protection by WR-2721. *Cancer* **61**: 2192–2195

Montana GS, Anscher MS, Mansbach II CM, Daly N, Delannes M, Clarke-Pearson D and Gaydica F (1992) Topical application of WR-2721 to prevent radiation-induced proctosigmoiditis. *Cancer* **69**: 2826–2830

Mori T, Nikaido O and Sugahara T (1984) Dephosphorylation of WR-2721 with mouse tissue homogenates. *Int J Radiat Oncol Biol Phys* **10**: 1529–1531

Murley JS and Grdina DJ (1995) The effects of cycloheximide and WR-1065 on radiation-induced repair processes: a mechanism for chemoprevention. *Carcinogenesis* **16**: 2699–2705

Murley JS, Constantinou A, Kamath NS and Grdina DJ (1997) WR-1065, an active metabolite of the cytoprotector amifostine, affects phosphorylation of topoisomerase II alpha leading to changes in enzyme activity and cell cycle progression in CHO AA8 cells. *Cell Profil* **30**: 283–294

Nagy B and Grdina DJ (1986) Protective effects of WR-2721 against bleomycin and nitrogen mustard-induced mutagenicity in V76 cells. *Int J Radiat Oncol Biol Phys* **12**: 1475–1478

Nagy B, Dale PJ and Grdina DJ (1986) Protection against cis diamminedichloroplatinum cytotoxicity and mutagenicity in V79 cells by WR-2721. *Cancer Res* **46**: 1132–1135

Nooijen PT, Westphal JR, Eggermont AM, Schalkwijk C, Max R, De Waal RM and Ruiter DJ (1998) Endothelial P-selectin expression is reduced an advanced primary melanoma and melanoma metastasis. *Am J Pathol* **152**: 679–682

Parnis FX, Coleman RE, Harper PG, Pickering D, Topham C, Whittington JR and Tedeschi M (1995) A randomised double-blind placebo controlled clinical trial assessing the tolerability and efficacy of glutathione as an adjuvant to escalating doses of cisplatin in the treatment of advanced ovarian cancer. *Eur J Cancer* **31A**: 1721

Planting AST, Vermorken JB, Catimel G, De Mulder PHM, De Graeff A, Oster W, and Hoppener F (1996) Randomized phase II study of cisplatin with and without amifostine in patients with advanced head and neck cancer. *Proc Am Soc Clin Oncol* **15**: abstract # 887

Poplin EA, LoRusso P, Lokich JJ (1994) Randomized clinical trial of mitomycin-C with or without pretreatment with WR-2721 in patients with advanced colorectal cancer. *Cancer Chemother Pharmacol* **33**: 415–419

Romero FJ, Segura-Aguilar J, Monsalre E, Hermenegildo C, Nies E, Puertas FJ and Roma J (1990) Antioxidant and glutathione-related enzymatic activities in rat sciatic nerve. *Neurotoxicol Teratol* **12**: 603–605

Rubin JS, Wadler S, Beitler JJ, Haynes H, Rozenblit A, McGill F, Goldberg G and Runowicz C (1995) Audiological findings in a phase I protocol investigating the effect of WR-2721, high-dose cisplatin and radiation therapy in patients with locally advanced cervical carcinoma *J Laryngol Otol* **109**: 744–747

Sajjan AR, Hinchigeri SB and Datta KS (1991) Multiple forms of γ-glutamyl transpeptidase in human submandibular gland. *Clin Chim Acta* **197**: 133–139

Schiller JH, Storer B and Berlin J (1996) Amifostine, cisplatin and vinblastine in metastatic non-small-cell lung cancer; a report of high response rates and prolonged survival. *J Clin Oncol* **14**: 1913–1921

Schwindt EA, Collet AM, Mendez AE, Cabrini RL and Itoiz ME (1992) The first normal oral epithelium which γ-glutamyl transpeptidase activity has been detected. *Histochem J* **24**: 964–968

Shapiro CL and Henderson IC (1994) Late cardiac effects of adjuvant therapy: too soon to tell? *Ann Oncol* **5**: 196–198

Shaw LM, Turrisi AT and Glover DJ (1986) Human pharmacokinetics of WR-2721. *Int J Radiat Oncol Biol Phys* **12**: 1501–1504

Shaw LM, Bonner H, Nakashima H and Lieberman R (1994) Evidence for saturable metabolism. *Proc Am Soc Clin Oncol* **13**: abstract # 144

Shpall EJ, Stemmer SM, Hami L, Franklin WA, Shaw L, Bonner HS, Bearman SI, Peters WP, Bast RC, McCulloch W, Capizzi R, Mitchell E, Schein PS and Jones RB (1994) Amifostine (WR-2721) shortens the engraftment period of 4-hydroperoxy-cyclophosphamide-purged bone marrow in breast cancer patients receiving high-dose chemotherapy with autologous bone marrow support. *Blood* **83**: 3132–3137

Smoluk GD, Fahey RC, Calabro-Jones PM, Aguilera JA and Ward JF (1988) Radioprotection of cells in culture by WR-2721 and derivatives: form of the drug responsible for protection. *Cancer Res* **48**: 3641–3647

Smyth I, Bowman A, Perren T, Wilkinson P, Prescott R, Quinn KJ and Tedeschi M (1997) Glutathione reduces the toxicity and improves quality of life of women diagnosed with ovarian cancer treated with cisplatin: Results of a double-blind randomised trial. *Ann Oncol* **8**: 569–573

Steinherz LJ and Yahalom J (1993) Cardiac complications of cancer therapy. In *Cancer Principles & Practice of Oncology*, De Vita VT, Hellman S and Rosenberg SA (eds), pp. 2370–2385. Lippincott: Philadelphia

Swain SM, Whaley FS, Gerber MC, Weisberg S, York M, Spicer D, Jones SE, Wadler S, Desai A, Vogel C, Speyer J, Mittelman A, Reddy S, Pendergrass K, Velez-Garcia E, Ewer MS, Bianchine JR and Gams RA (1997) Cardioprotection with dexrazoxane for doxorubicin-containing therapy in advanced breast cancer. *J Clin Oncol* **15**: 1318–1332

Taylor CW, Wang LM, List AF, Fernandes D, Paine-Murrieta GD, Johnson CS and Capizzi RL (1997) Amifostine protects normal tissues from paclitaxel toxicity while cytotoxicity against tumour cells is maintained. *Eur J Cancer* **33**: 1693–1698

Thompson DC, Wyrick SD, Holbrook DJ and Chaney SG (1995) HPLC and $^{31}$P NMR characterization of the reaction between antitumor platinum agents and the phosphorothioate chemoprotective agent S-2-(3-aminopropylamino)ethylphosphorothioic acid (WR-2721). *Biochem Pharmacol* **50**: 1413–1419

Treskes M, Holwerda U, Klein I, Pinedo HM and Van der Vijgh WJF (1991) The chemical reactivity of the modulating agent WR-2721 (ethiofos) and its main metabolites with the antitumor agents cisplatin and carboplatin. *Biochem Pharmacol* **42**: 2125–2130

Treskes M, Boven E, Van de Loosdrecht AA and Wijffels JFAM (1994) Effects of the modulating agent WR-2721 on myelotoxicity and antitumour activity in carboplatin-treated mice. *Eur J Cancer* **30A**: 183–187

Utley JF, Marlowe C and Waddell WJ (1976) Distribution of 35S-labeled WR-2721 in normal and malignant tissues of the mouse. *Radiat Res* **68**: 284–291

Valeriote F and Tolen S (1982) Protection and potentiation of nitrogen mustard cytotoxicity by WR-2721. *Cancer Res* **42**: 4330–4331

Van der Vijgh WJ and Korst AE (1996) Amifostine (Ethyol): pharmacokinetic and pharmacodynamic effects in vivo. *Eur J Cancer* 32A(4): S26–S30.

Washburn LC, Rafter JJ, Hayes RL and Yuhas JM (1976) Prediction of the effective radioprotective dose of WR-2721 in humans through an interspecies tissue distribution study. *Radiat Res* **66**: 100–105

Wasserman TH, Philips TL, Ross G and Kane LJ (1981) Differential protection against cytotoxic chemotherapeutic effects on bone marrow CFUs by WR-2721. *Cancer Clin Trials* **4**: 3–6

Weiss JF (1997) Pharmacologic approaches to protection against radiation-induced lethality and other damage. *Environ Health Perspect* **105S** (suppl 6): 1473–1478

© Cancer Research Campaign 1999

Wooley PV, Ayoob MJ and Smith FP (1983) Clinical trial of the effect of S-2(3-aminopropylamino)-ethylphosphorothioic acid (WR-2721) (NSC 296961) on the toxicity of cyclophosphamide. *J Clin Oncol* **5**: 198–203
Yuhas JM (1980) Active versus passive absorption kinetics as the basis for selective protection of normal tissues by S-2-(3-aminopropylamino)-ethylphosphorothioic acid. *Cancer Res* **40**: 1519–1524
Zunino F, Prates G, Micheloni A, Cavalleti E, Sala F and Tofanetti O (1989) Protective effect of reduced glutathione against cisplatin induced renal and systemic toxicity and its influence on the therapeutic activity of the anti-tumour drug. *Chem Biol Interact* **70**: 89–101

*© Cancer Research Campaign 1999*
